# Tumor CTLA-4 overexpression predicts poor survival in patients with nasopharyngeal carcinoma

**DOI:** 10.18632/oncotarget.7421

**Published:** 2016-02-16

**Authors:** Pei-Yu Huang, Shan-Shan Guo, Yu Zhang, Jia-Bin Lu, Qiu-Yan Chen, Lin-Quan Tang, Lu Zhang, Li-Ting Liu, Li Zhang, Hai-Qiang Mai

**Affiliations:** ^1^ State Key Laboratory of Oncology in South China, Collaborative Innovation Center for Cancer Medicine, Sun Yat-Sen University Cancer Center, Guangzhou, China; ^2^ Department of Nasopharyngeal Carcinoma, Sun Yat-Sen University Cancer Center, Guangzhou, China; ^3^ Department of Pathology, Sun Yat-Sen University Cancer Center, Guangzhou, China; ^4^ Department of Medical Oncology, Sun Yat-Sen University Cancer Center, Guangzhou, China

**Keywords:** CTLA-4, CD28, nasopharyngeal carcinoma, immunohistochemistry, prognostic factor

## Abstract

The expression levels of CTLA-4 and CD28 were analyzed in 191 nasopharyngeal carcinoma (NPC) patients diagnosed and treated at our hospital between January 2010 and November 2011. The 3-year overall survival (OS) rate (91.4% vs. 81.2%,*p* = 0.043), failure-free survival (FFS) rate (82.8% vs. 68.0%, *p* = 0.009) and distant failure-free survival (D-FFS) rate (85.8% vs. 72.3%, *p* = 0.006) in the low tumor CTLA-4 expression group was higher than in the high tumor CTLA-4 group. There were no differences between the locoregional failure-free survival (LR-FFS) rates in the high and low tumor CTLA-4 expression groups. Moreover, no differences in the OS, FFS, D-FFS, or LR-FFS were observed between the groups with high and low lymphocyte CTLA-4 levels, high and low tumor CD28 levels, or high and low lymphocyte CD28 levels. Cox regression analysis confirmed the prognostic value of tumor CTLA-4 expression, particularly for D-FFS, in NPC patients (*p* = 0.044). NPC patients with high tumor CTLA-4 expression had a poorer prognosis than those with low expression.

## INTRODUCTION

Nasopharyngeal carcinoma (NPC) is endemic in southern China and South-East Asia. There are approximately 80,000 incident cases and 50,000 deaths annually worldwide, but there are remarkable variations in the racial and geographic distributions [1]. Radiotherapy (RT), particularly intensity-modulated radiotherapy (IMRT), is the recommended treatment for non-metastatic disease [2]. Concurrent chemoradiotherapy (CCRT) with or without adjuvant chemotherapy (AC) is the primary regimen for patients with locoregionally advanced NPC [3, 4]. However, patients with similar stages and histological classifications have different survival outcomes due to the heterogeneity of the tumor protein expression profiles. The development of novel tumor markers to stratify treatment outcomes might enable better prediction of patient prognosis, provide insight into the mechanisms responsible for treatment failure, and result in the identification of novel therapeutic targets.

The cytotoxic T-lymphocyte associated antigen-4 (CTLA-4) is a well-known activator of T cells [5, 6]. CTLA-4 is expressed on the surface of T cells upon activation and interacts with B7 ligands (CD80/CD86) expressed on antigen presenting cells to inhibit cell proliferation, cytokine (interleukin-2 and interferon) production, and cell cycle progression [7, 8]. CD28 is a major T cell co-stimulatory receptor, the co-engagement of which can prevent anergy and cell death [9].

The prognostic role of CTLA4 has been explored in several types of cancers. For example, CTLA4 overexpression was detected in non-squamous type non-small cell lung cancer and was associated with a reduced death rate [10]. Breast cancer patients with higher CTLA-4 mRNA levels had obvious axillary lymph node metastases and a higher clinical stage [11]. CTLA4 downregulation led to a significant increase in the proliferation and survival of chronic lymphocytic leukemia cells [12]. CD28 expression has been reported to correlate with tumor progression in multiple myeloma [13, 14]. However, no studies have addressed the prognostic value of CTLA4 and CD28 expression in NPC.

In the present study, we recruited NPC patients who were treated with cisplatin-based CCRT and investigated the expression of CTLA4 and CD28 in NPC tissue. We hypothesized that the expression of CTLA4 and CD28 could be of potential prognostic value for patients with NPC.

## RESULTS

### Patient characteristics and immunohistochemical analysis

The study included 44 women (44 of 191 patients, 23.0%) and 147 men (147 of 191 patients, 77.0%) with a median age of 50 years (range 19–79). Five (2.6%) patients were classified as stage I, 22 (11.5%) as stage II, 78 (40.8%) as stage III, 74 (38.7%) as stage IVa or IVb, and 12 (6.3%) as stage IVc. Six (3.1%) patients were classified as World Health Organization (WHO) II and 185 (96.9%) as WHO III.

**Figure 1:**
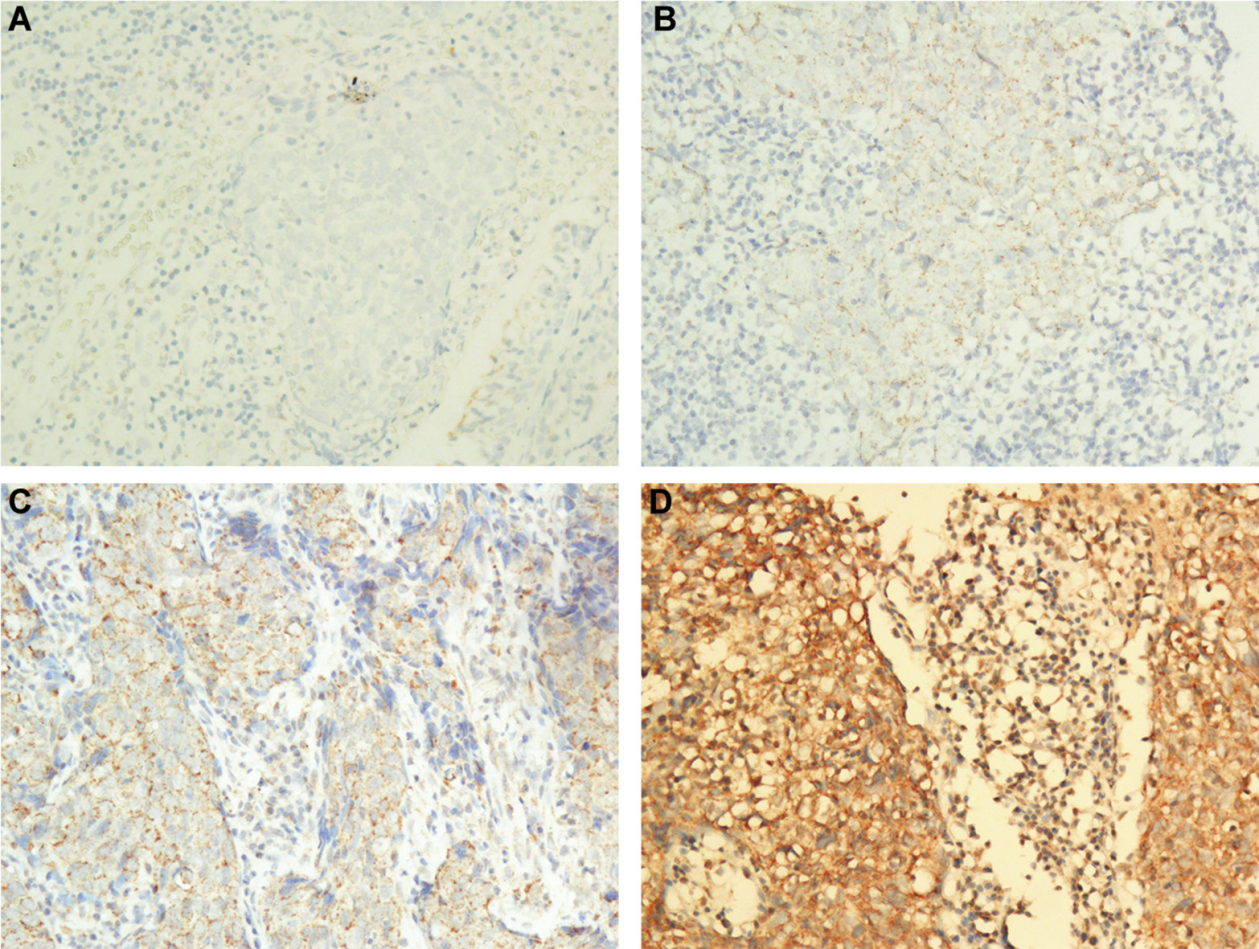
Representative images of the different intensities of the IHC staining for CTLA-4 expression (**A**) Negative staining of CTLA-4 in NPC tumor and lymphocyte (200 ×); (**B**) Weak staining of CTLA-4 in NPC tumor and lymphocyte (200 ×); (**C**) Moderate staining of CTLA-4 in NPC tumor and lymphocyte (200 ×); (**D**) Strong staining of CTLA-4 in NPC tumor and lymphocyte (200 ×).

**Figure 2:**
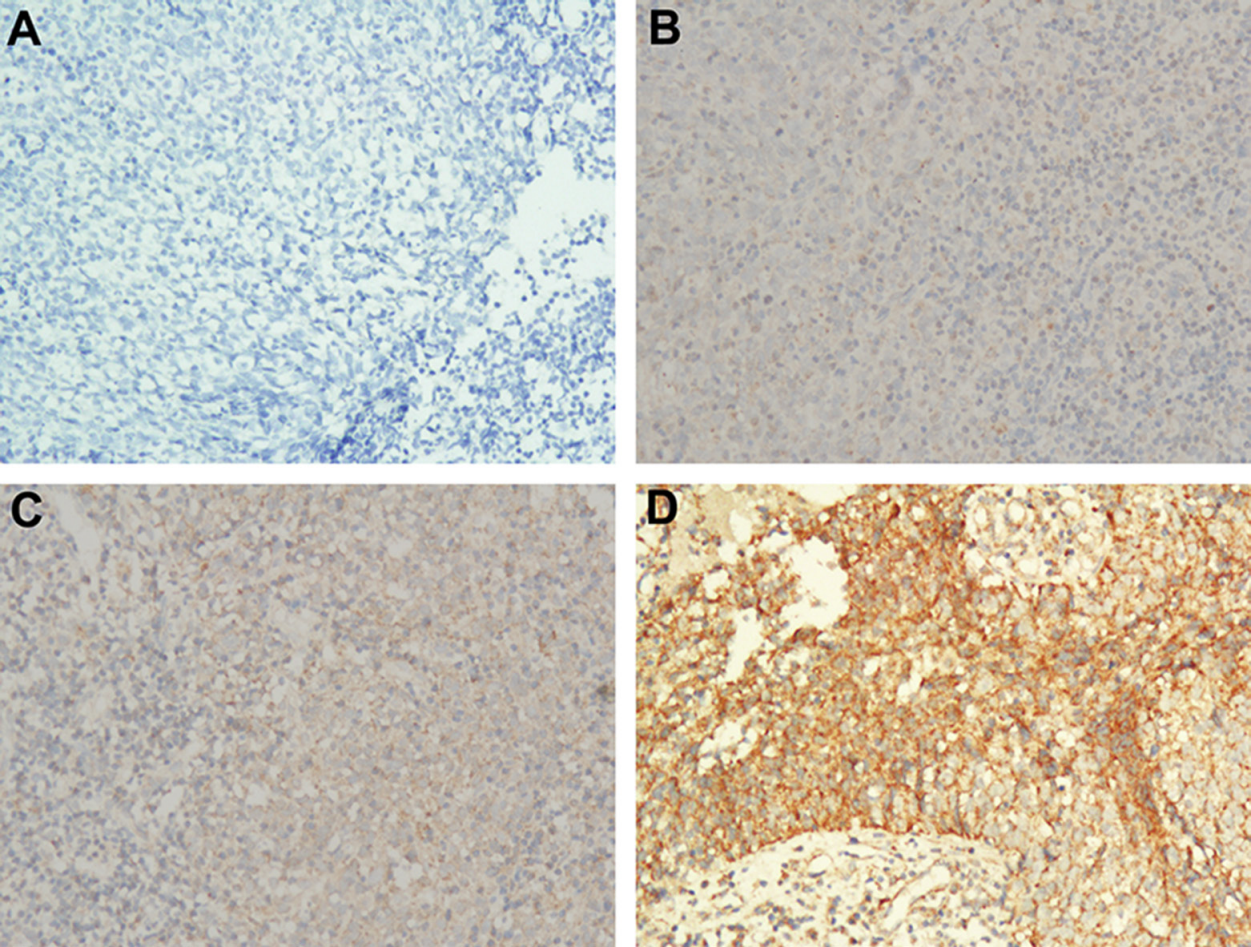
Representative images of the different intensities of IHC staining for CD28 expression (**A**) Negative staining of CD28 in NPC tumor and lymphocyte (200 ×); (**B**) Weak staining of CD28 in NPC tumor and lymphocyte (200 ×); (**C**) Moderate staining of CD28 in NPC tumor and lymphocyte (200 ×); (**D**) Strong staining of CD28 in NPC tumor and lymphocyte (200 ×).

CTLA-4 expression with different intensities in the tumor cell cytoplasm (Figure 1) was observed in 186 (97.4%) patients. CTLA-4 expression with different intensities in the lymphocyte cytoplasm (Figure 1) was observed in 185 (96.9%) patients. Finally, CD28 expression with different intensities in the tumor cell cytoplasm (Figure 2) was observed in 187 (97.9%) patients, and CD28 expression with different intensities in the lymphocyte cytoplasm (Figure 2) was observed in 178 (93.2%) patients.

The final CTLA-4 or CD28 score was calculated as the proportion score × staining intensity. The median tumor CTLA-4 score was 2, the median lymphocyte CTLA-4 score was 0.7, the median tumor CD28 score was 1, and the median lymphocyte CD28 score was 0.7. When the CTLA-4 score of the tumor was 2 or higher, patients were considered to have high expression (present in 98 of 191 patients). When the CTLA-4 score of the lymphocytes was 0.7 or higher, the patients were considered to have high expression (present in 101 of 191 patients). A CD28 score of the tumor greater than or equal to 1 was observed in 126 of 191 patients, and these patients were considered to have high expression. The CD28 score of the lymphocytes in 96 of 191 patients with detec table expression was greater than or equal to 0.7, and these patients were assigned to the high expression group. In the present study, the baseline characteristics of the CTLA-4 or CD28 high and low expression groups are shown in Table 1.

**Table 1:** Baseline characteristics of the patients in the different CTLA-4 and CD28 groups

	Tumor CTLA-4 low expression(*n* = 93)	Tumor CTLA-4 high expression(*n* = 98)	*p* value	Lymphocyte CTLA-4 low expression(*n* = 90)	Lymphocyte CTLA-4 high expression(*n* = 101)	*p* value	Tumor CD28 low expression(*n* = 65)	Tumor CD28 high expression(*n* = 126)	*p* value	Lymphocyte CD28 lowe xpression(*n* = 95)	Lymphocyte CD28 high expression(*n* = 96)	*p* value
Age [mean (range)]	47.5 (19–79)	50.4 (20–78)	0.115	48.6 (19–79)	49.3 (20–78)	0.704	48.7 (25–79)	49.5 (19–76)	0.824	48.4 (20–79)	49.6 (19–76)	0.498
Age												
≥ 50	41	55	0.096	41	55	0.219	33	63	0.920	47	49	0.828
*<* 50	52	43		49	46		32	63		48	47	
Sex			0.219			0.261			0.462			0.157
Male	68 (73.1%)	79 (80.6%)		66 (73.3%)	81 (80.2%)		48 (73.8%)	99 (78.6%)		69 (72.6%)	78 (81.3%)	
Female	25 (26.9%)	19 (19.4%)		24 (26.7%)	20 (19.8%)		17 (26.2%)	27 (21.4%)		26 (27.4%)	18 (18.8%)	
Stage			0.047			0.803			0.088			0.225
I II III IVa or IVb	4 (4.3%) 13 (14.0%) 39 (41.9%) 34 (36.6%)	1 (1.0%) 9 (9.2%) 39 (39.8%) 40 (40.8%)		1 (1.1%) 11 (12.2%) 39 (43.3%) 34 (37.8%)	4 (4.0%) 11 (10.9%) 39 (38.6%) 40 (39.6%)		1 (1.5%) 11 (16.9%) 29 (44.6%) 21 (32.3%)	4 (3.2%) 11 (8.7%) 49 (38.9%) 53 (42.1%)		2 (2.1%) 13 (13.7%) 42 (44.2%) 32 (33.7%)	3 (3.1%) 9 (9.4%) 36 (37.5%) 42 (43.8%)	
IVc	3 (3.2%)	9 (9.2%)		5 (5.6%)	7 (6.9%)		3 (4.6%)	9 (7.1%)		6 (6.3%)	6 (6.3%)	
WHO type			0.727			0.003			0.218			0.399
II III	2 (2.2%) 91 (97.8%)	4 (4.1%) 94 (95.9%)		0 90 (100.0%)	6 (5.9%) 95 (94.1%)		3 (4.6%) 62 (95.4%)	3 (2.4%) 123 (97.6%)		4 (4.2%) 91 (95.8%)	2 (2.1%) 94 (97.9%)	

Abbreviations: WHO = World Health Organization.

The association study showed that the expression of CTLA-4 in the tumor was significantly associated with the UICC stage. The low tumor CTLA-4 expression group had more early stage (stage I–II) patients than the CTLA-4 high expression group (18.3% vs. 10.2%, *p* = 0.047). Moreover, the low lymphocyte CTLA-4 expression group had fewer WHO II patients than the CTLA-4 high expression group (0% vs. 5.9%, *p* = 0.003). The association study showed that CTLA-4 or CD28 expression in the tumor or lymphocytes was not significantly associated with any of the other clinicopathological features examined including age, gender, UICC stage or WHO pathological type of the patients (Table 1).

### Correlation between CTLA-4 or CD28 expression in the tumor or lymphocytes and the clinical outcomes of NPC patients

**Figure 3:**
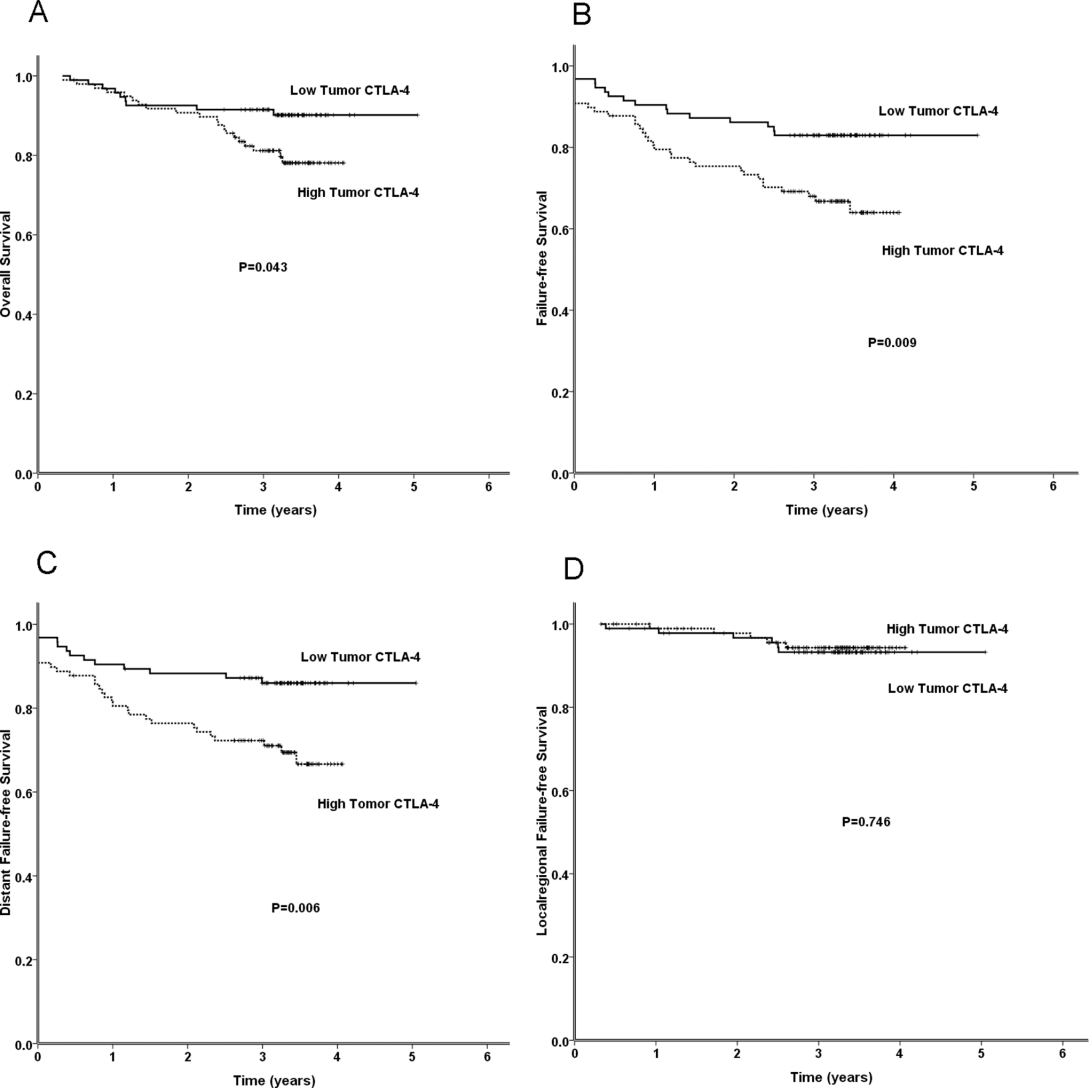
The results of a comparison between the low and high tumor CTLA-4 expression groups with regard to the OS, FFS, D-FFS, and LR-FFS rates

The 3-year overall survival (OS), failure-free survival (FFS), locoregional failure-free survival (LR-FFS) and distant failure-free survival (D-FFS) rates for the CTLA-4 or CD28 high and low expression groups are shown in Tables 2 and 3. The 3-year OS in the low tumor CTLA-4 expression group was higher than that in the high tumor CTLA-4 expression group (91.4% vs. 81.2%, *p* = 0.043) (Figure 3A). The 3-year FFS in the low tumor CTLA-4 expression group was also higher than that in the high tumor CTLA-4 expression group (82.8% vs. 68.0%, *p* = 0.009) (Figure 3B). Moreover, the 3-year D-FFS in the low tumor CTLA-4 expression group was higher than that in the high tumor CTLA-4 expression group (85.8% vs. 72.3%, *p* = 0.006) (Figure 3C). There were no significant differences in the LR-FFS between the high and low tumor CTLA-4 expression groups. In addition, no significant differences in the OS, FFS, D-FFS, or LR-FFS were observed between the high and low lymphocyte CTLA-4 expression groups, high and low tumor CD28 expression groups, or high and low lymphocyte CD28 expression groups (Tables 2 and 3).

**Table 2:** The different survival results in the different tumor CTLA-4 and lymphocyte CTLA-4 expression groups

	Tumor CTLA-4			Lymphocyte CTLA-4		
	Low expression	High expression	*P* value	Low expression	High expression	*P* value
3-year OS	91.4%	81.2%	0.043	90.0%	82.7%	0.300
3-year FFS	82.8%	68.0%	0.009	79.9%	71.1%	0.091
3-year D-FFS	85.8%	72.3%	0.006	84.2%	74.1%	0.077
3-year LR-FFS	93.2%	94.3%	0.746	92.9%	94.6%	0.668

Abbreviations: OS, overall survival; FFS, failure free survival; D-FFS, distant failure-free survival; LR-FFS, localregional failure-free survival.

**Table 3:** The different survival results in the different tumor CD28 and lymphocyte CD28 expression groups

	Tumor CD28			Lymphocyte CD28		
	Low expression	High expression	*P* value	Low expression	High expression	*P* value
3-year OS	85.7%	86.5%	0.695	87.1%	85.3%	0.779
3-year FFS	76.4%	73.7%	0.752	75.5%	73.8%	0.849
3-year D-FFS	82.8%	76.9%	0.505	78.6%	77.9%	0.812
3-year LR-FFS	91.7%	94.8%	0.409	94.3%	93.2%	0.777

Abbreviations: OS, overall survival; FFS, failure free survival; D-FFS, distant failure-free survival; LR-FFS, localregional failure-free survival.

In the present study, a Cox regression analysis (Table 4) showed that the UICC stage was a significant prognostic factor that affected OS (*p* = 0.030). Body mass index (BMI) and C-reactive protein (CRP) level were also significant prognostic factors (*p* < 0.001 and *p* = 0.001, respectively). Tumor CTLA-4 expression was a marginally significant prognostic factor for FFS (*p* = 0.066). The UICC stage was a significant prognostic factor (*p* < 0.001) and the CRP level was a significant prognostic factor (*p* = 0.022). Tumor CTLA-4 expression was a significant prognostic factor for D-FFS (*p* = 0.044), and the UICC and CRP level were also significant prognostic factors (*p* < 0.001 and *p* = 0.005, respectively). No significant prognostic factors were found for LR-FFS. In addition, lymphocyte CTLA-4 expression (Supplementary Table 1), tumor CD28 expression (Supplementary Table 2), and lymphocyte CD28 expression (Supplementary Table 3) were not significant prognostic factors for OS, FFS, D-FFS, or LR-FFS.

**Table 4:** The significant factors associated with the survival of NPC patients identified in a multivariate analysis that included the tumor CTLA-4 expression

Characteristics	OS			FFS			D-FFS			LR-FFS		
	HR	95% CI	*P value*	HR	95% CI	*P value*	HR	95% CI	*P value*	HR	95% CI	*P value*
Tumor CTLA-4	2.069	0.929–4.606	0.075	1.769	0.964–3.247	0.066	1.992	1.020–3.891	0.044	*		
BMI	0.762	0.661–0.879	0.000	0.914	0.828–1.008	0.071	*			0.819	0.665–1.008	0.059
UICC stage	1.765	1.057–2.948	0.030	5.207	2.849–9.518	0.000	6.528	3.370–12.649	0.000	2.029	0.906–4.544	0.085
CRP	1.047	1.020–1.075	0.001	1.027	1.004–1.050	0.022	1.032	1.009–1.055	0.005	*		

Abbreviations: OS, overall survival; FFS, failure free survival; D-FFS, distant failure-free survival; LR-FFS, localregional failure-free survival.* Not a significant factor.

## DISCUSSION

NPC is one of the Epstein-Barr virus (EBV)-associated malignancies with distinct epidemiology, etiology, and clinical biological behavior compared to other head and neck cancers [15]. Identifying the patients who have the potential for immune escape and a greater risk of primary treatment failure is very important. In the present study, the co-expression of CD28 and CTLA-4 on both tumor cells and tumor-infiltrating lymphocytes (TILs) was detected. Furthermore, the impact of CD28 and CTLA-4 expression together with various clinical parameters on the survival of a cohort of NPC patients was assessed. To the best of our knowledge, this is the first study to explore the prognostic value of the co-expression of these two immune markers in NPC patients.

The expression of immunosuppressive proteins in cancer appears to help the tumor escape host immune surveillance, while the expression of immunosuppressive proteins in the immune cells around the tumor appears to be a host response to the tumor [16]. However, the clinical significance of the existence of immunosuppressive proteins in both tumors and immune cells in the tumor microenvironment is still controversial, and their potential as prognostic markers and therapeutic targets needs to be investigated.

CLTA-4, one of the most important immunosuppressive proteins that acts via interactions with its ligands CD80 and CD86, plays a key role in attenuating the early activation of naïve and memory T cells [17]. Kim et al. [16] found that in gastric cancer, the expression of immunosuppressive proteins, including PD-L1, CTLA-4, and IDO, in tumors was associated with less advanced stage, intestinal type, and well/moderately differentiated adenocarcinoma. Although there was no statistical significance, better prognoses were also noted for gastric cancer patients with CTLA-4 expression in the tumors. A CTLA-4 polymorphism analysis conducted by Xiao et al. [18] showed that CTLA-4 single nucleotide polymorphisms were highly associated with NPC susceptibility in a Chinese population. However, no previous studies have examined whether CTLA-4 expression influences the prognosis of NPC patients.

CD28 is normally expressed on 95% of CD4+ T cells and approximately 50% of CD8+ T cells in human peripheral blood [19]. It plays a key role as a co-stimulatory signal during antigen/major histocompatibility complex presentation [20]. Decreased CD28 expression has been observed in some types of cancer, for example, in the dysfunctional peripheral T-lymphocytes from patients with hairy cell leukemia [21] and chronic lymphocytic leukemia [22], as well as in colorectal cancer patients [23]. However, whether CD28 expression on TILs or tumor cells influences the prognosis of NPC patients has not been evaluated.

In the present study, we found that the 3-year OS, FFS, and D-FFS in the low tumor CTLA-4 expression group were significantly higher than those in the high tumor CTLA-4 expression group, although the low tumor CTLA-4 expression group had more early stage (stage I–II) patients than the high CTLA-4 expression group (18.3% vs. 10.2%, *p* = 0.047). After adjusting for the UICC stage and other important factors including gender, age, smoking status, first-degree family history of NPC, BMI, and CRP level, a Cox regression analysis confirmed the prognostic value of the tumor CTLA-4 expression, particularly the D-FFS, in NPC patients. However, no significant association between CTLA-4 expression on TILs and clinical outcomes was observed. Additionally, there was no apparent association between CD28 expression on tumor cells or TILs and clinical outcomes.

T cell activation and suppression require the interaction between B7 on an antigen-presenting cell and CD28 or CTLA-4 on a T cell. Immediately after activation, CTLA-4 translocates to the plasma membrane where it downregulates the functions of T cells to maintain immunological homeostasis [24]. Pistillo et al. [25] found that CTLA-4 was not restricted to the lymphoid cell lineage and could be a target to induce apoptosis in leukemic cells. In acute B and T cell leukemias, the CTLA-4 expression was mainly cytoplasmic, while in chronic B cell leukemias, it was expressed on both the cell surface and in the cytoplasm. Chronic T-cell leukemias were found to be negative for CTLA-4 in a few cases. In solid tumors, cytoplasmic and surface CTLA-4 expression was detected in all six osteosarcoma specimens and in all five cases of ductal breast carcinomas examined. In gastric cancer [16], CTLA-4 was expressed in the cytoplasm of tumor cells. In the present study of NPC, CTLA-4 expression was found not only in TILs but also in tumor cells. In NPC cells, CTLA-4 was almost exclusively expressed in the cytoplasm. This was also true of the TILs in NPC, which is consistent with the fact that the majority of CTLA-4 is localized in vesicles of the Golgi apparatus and is released to the cell surface during T cell activation [15, 26]. Only small amounts of CTLA-4 can be detected on the cell surface at any given time, even following T-cell activation [26].

The intracellular trafficking pathways that control the transport of CTLA-4 to the cell surface influence the degree of inhibition and the potency of antibody checkpoint blockade in cancer immunotherapy. This mechanism may at least partly explain why the density and intensity of CTLA-4 expression in the cytoplasm of the TILs did not have a significant correlation with the prognosis of NPC patients in our study.

Recent studies have confirmed the survival benefit of ipilimumab (a monoclonal antibody that targets CTLA-4) in patients with advanced melanoma [27] and non-small-cell lung cancer [28]. However, the potential therapeutic effects of ipilimumab against other solid tumors such as NPC have not been investigated. At present, although no single immunological or tumor-related factor has been found to solely determine the response to an immunotherapeutic agent [29], the expression of CTLA-4 in the tumor or TILs may represent a target, and CTLA-4 blockade may provide therapeutic benefits for NPC.

Although we did not find any correlation between the lymphocyte CTLA-4 expression and NPC patient clinical outcome in our study, we did find that the OS, FFS, and D-FFS rates in the low tumor CTLA-4 expression group were significantly higher than those in the high tumor CTLA-4 expression group. A Cox regression analysis confirmed the prognostic value of the tumor CTLA-4 expression in NPC patients, especially for the D-FFS of NPC patients.

In conclusion, NPC patients with high tumor CTLA-4 expression had a poor prognosis. This group of patients might be ideal for a clinical trial of anti-CTLA-4 therapy.

## MATERIALS AND METHODS

### Ethics statement

The Institutional Review Board of Sun Yat-Sen Cancer Center approved this study protocol. Written informed consent was obtained from patients for the collection of the tissue samples.

### Patient recruitment and follow–up

The expression of CTLA-4 and CD28 in tumor cells was evaluated in tumor samples obtained before treatment from 191 previously untreated, histologically confirmed, stage I–IVc (according to the 7th edition of the AJCC/UICC criteria), WHO II–III NPC patients who were prospectively enrolled between January 2010 and November 2011. Among the enrolled patients, 147 were male and 44 were female, with a sex ratio of 3.3:1. The median age of the patients was 50 years (range, 19–79 years). The radiation technique used was IMRT, which was performed in accordance with the treatment policy for NPC at Sun Yat-sen University Cancer Center. For non-metastatic disease, the treatment regimens included (a) RT alone, (b) CCRT, (c) induction chemotherapy (IC) + CCRT, and (d) CCRT + AC. The regimen used for IC was PF (cisplatin 80–100 mg/m^2^ intravenously (IV) on day 1 and 5-Fu 800 mg/m^2^/d continuously IV on days 1–5). The treatment regimens were repeated every 3 weeks for two to three cycles. Concurrent chemotherapy primarily consisted of the following two regimens: cisplatin 80–100 mg/m^2^ IV every 3 weeks and cisplatin 30–40 mg/m^2^ IV weekly. The adjuvant chemotherapy regimen was PF (cisplatin 80 mg/m^2^ IV on day 1 and 5-Fu 800 mg/m^2^/d continuously IV on days 1–5). Adjuvant chemotherapy was repeated every 4 weeks for three cycles. Palliative chemotherapy was administered to patients with metastatic disease. After the completion of treatment, patients were followed up monthly for the first 3 months, every 3 months through 3 years, every 6 months for the next 2 years, and then annually thereafter. The median follow-up was 3.4 years (range, 0.3–5.1 years).

### Immunohistochemistry

Antibodies were subjected to in-house validation by the manufacturer for IHC analysis on paraffin-embedded material. The antibodies used in the study were CD152 (Rabbit; 251548; Abbiotec, San Diego, California, U.S.A.) and CD28 (Rabbit; 251660; Abbiotec). Tissue sections were deparaffinized with xylene and rehydrated with ethanol. Hydrogen peroxide (3%) was used to remove endogenous peroxidase. Antigen retrieval was performed in 0.01 mmol/L sodium citrate buffer (pH 6.0) for 5 min in a microwave. The samples were incubated with the primary antibodies for 30 minutes at 37°C. The sections were then washed with PBS and incubated with secondary antibodies (EnVision, Dako, Carpinteria, CA, U.S.A.) for 120 minutes at 37°C. The antigens were visualized with 3,3′-Diaminobenzidine. Sections were counterstained with hematoxylin. Hydrochloric acid alcohol was used for differentiation. Lithium carbonate was used to turn the slices back to blue.

### Scoring of the immunohistochemistry findings

The expression of CTLA4 and CD28 was scored by combining (a) the percentage of positively stained cells determined using light microscopy (a proportion score was assigned [i.e., tumors with 50% of the cytoplasm stained were assigned a score of 0.5]) with (b) the intensity of staining (0, negative staining; 1, mild staining; 2, moderate staining; 3, strong staining). The final score was assessed as a × b. All specimens were evaluated by two independent pathologists without prior knowledge of the clinical origin of the specimen. The values were accepted if the results reported by the pathologists were consistent. In cases for which the results were inconsistent, the pathologists worked to reach a compromise on the score.

### Statistical analysis

The Statistical Package for Social Sciences version 16.0 software program (SPSS Inc., Chicago, IL, U.S.A.) was used for the analysis. A chi-squared analysis was used to compare the incidence rates and categorical variables. The means of continuous variables were compared using Student's *t*-tests. The survival rates were calculated using the Kaplan-Meier method and were compared using log-rank tests. Multivariate analyses were performed using the Cox proportional hazards model. Hazard ratio point and interval (95% confidence interval) estimates were computed using the Cox proportional hazards model. The potentially important prognostic factors that were considered in the modeling process were the following: patient gender, age, smoking status, first-degree family history of NPC, BMI, UICC stage, CRP level, and CTLA-4 or CD28 expression in the tumor or lymphocytes.

The smoking status at diagnosis was categorized into three groups: (a) never-smokers, which referred to patients who had never smoked; (b) ex-smokers, which referred to former smokers who had stopped smoking, and (c) smokers who continued smoking until the day of the diagnosis of NPC. All of the *p* values were two-sided, and *p* < 0.05 was considered to be statistically significant.

## SUPPLEMENTARY MATERIALS FIGURES AND TABLES


