# Supplementary material for: Tumor CTLA-4 overexpression predicts poor survival in patients with nasopharyngeal carcinoma
Source: Oncotarget. 2016 Feb 16;7(11):13060–8. doi: 10.18632/oncotarget.7421 (PMC4914341; doi:10.18632/oncotarget.7421)
Supplement: Supplementary file 1 [file oncotarget-07-13060-s001.pdf]

Tumor CTLA-4 overexpression predicts poor survival in patients with nasopharyngeal carcinoma

Supplementary Materials

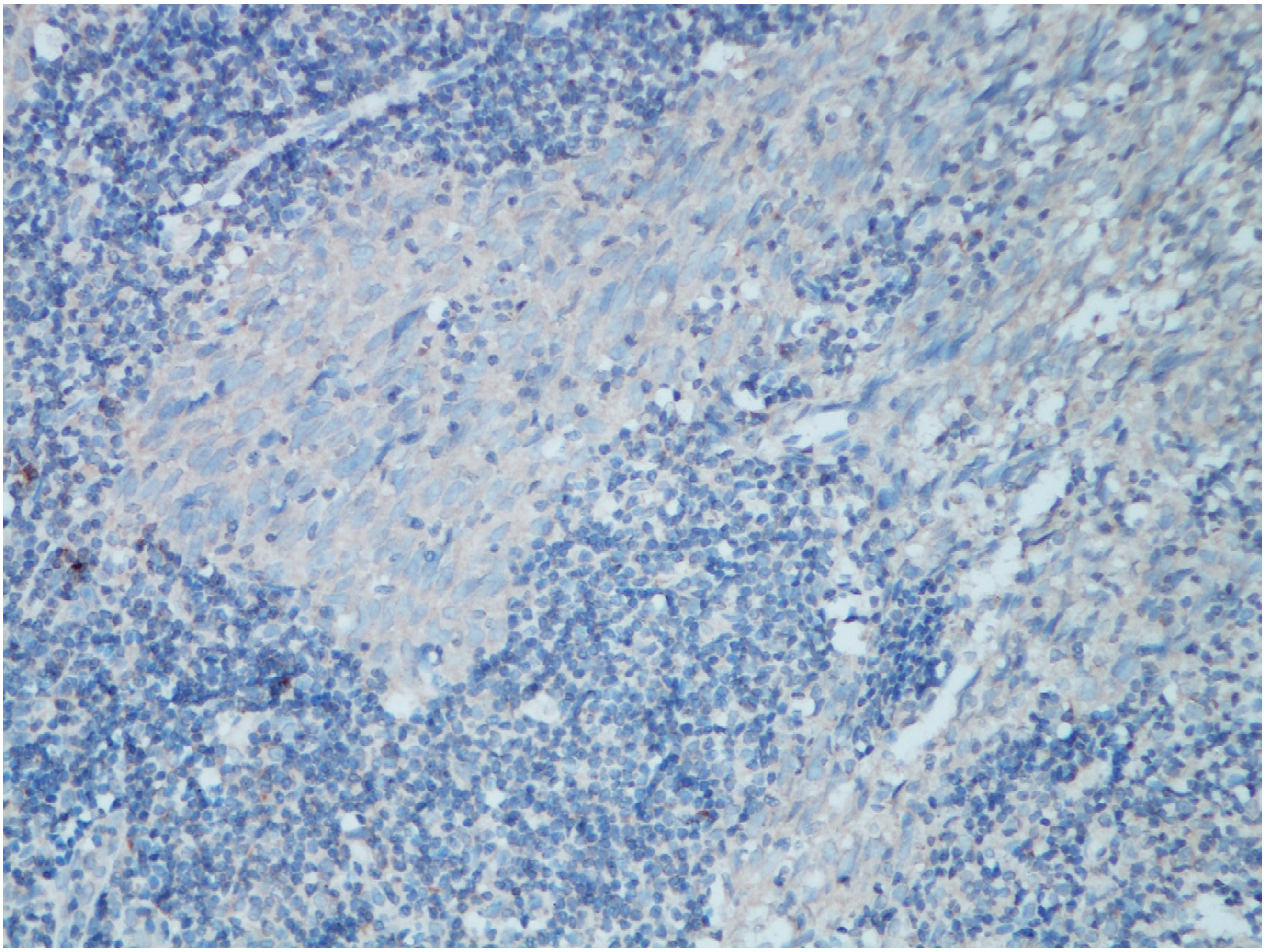

Supplementary Figure S1: The IHC staining for CTLA-4 expression (200 ×) using Santa cruz antibody.

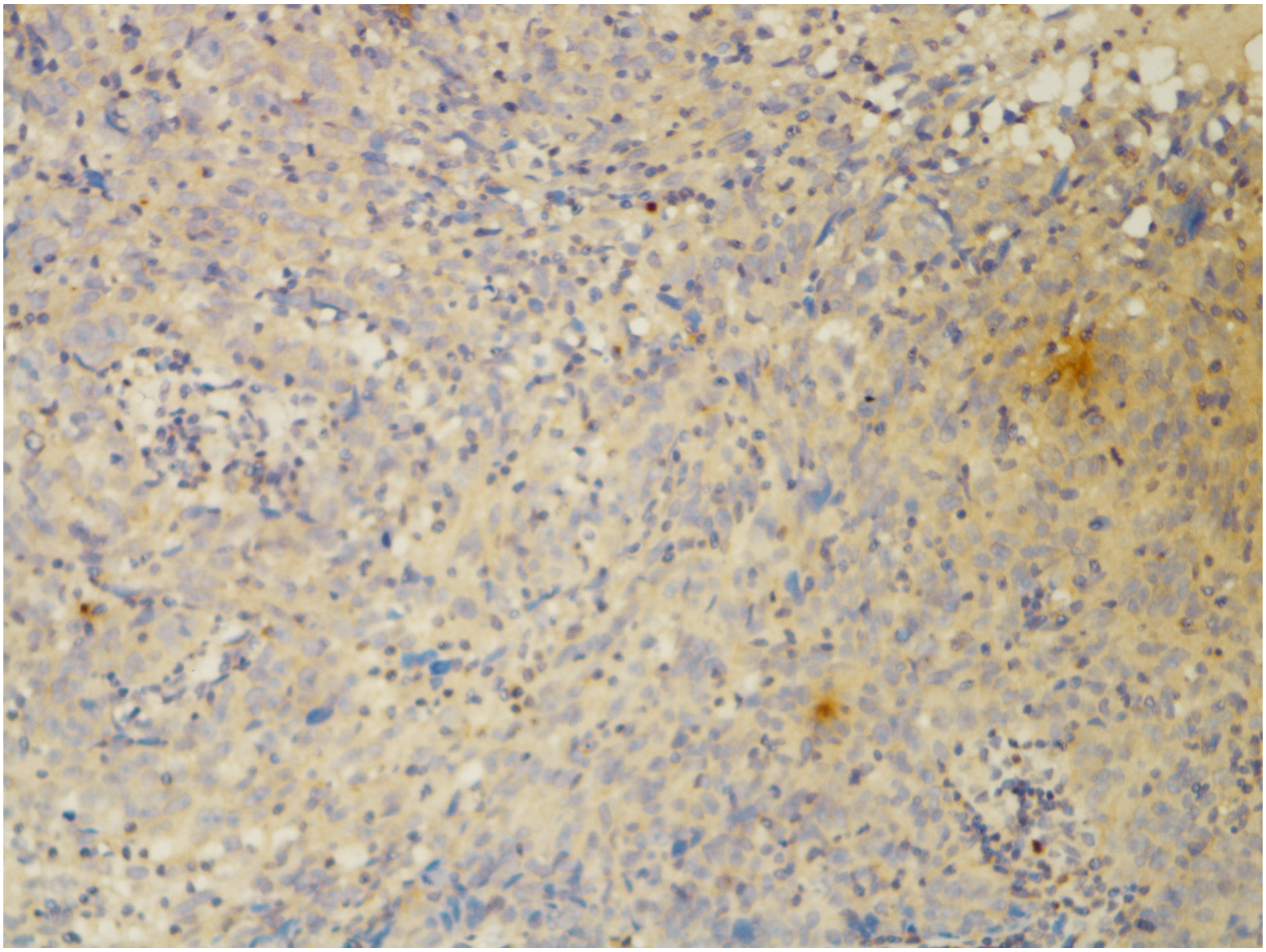

Supplementary Figure S2: The IHC staining for CD28 expression (200 ×) using abcam antibody.

Supplementary Table S1: Significant factors in multivariate analysis including lymphocyte CTLA-4 expression of different endpoint in Cox regression model

Characteristics	OS			FFS			D-FFS			LR-FFS		
	**HR**	**95% CI**	***P value***	**HR**	**95% CI**	***P value***	**HR**	**95% CI**	***P value***	**HR**	**95% CI**	***P value***
Lymphocyte CTLA-4	*			1.669	0.926–3.006	0.088	1.768	0.936–3.343	0.079	*		
BMI	0.775	0.675–0.888	0.000	0.909	0.825–1.001	0.053	0.913	0.822–1.013	0.087	0.819	0.665–1.008	0.059
UICC stage	1.886	1.144–3.107	0.013	5.307	2.899–9.716	0.000	6.422	3.264–12.632	0.000	2.029	0.906–4.544	0.085
CRP	1.045	1.019–1.071	0.001	1.026	1.003–1.049	0.027	1.031	1.008–1.053	0.007	*		

Abbreviations: OS, overall survival; FFS, failure free survival; D-FFS, distant failure-free survival; LR-FFS, localregional failure-free survival.* Not a significant factor.

Supplementary Table S2: Significant factors in multivariate analysis including tumor CD28 expression of different endpoint in Cox regression model

Characteristics	OS			FFS			D-FFS			LR-FFS		
	HR	95% CI	*P value*	HR	95% CI	*P value*	*HR*	95% CI	*P value*	HR	95% CI	*P value*
BMI	0.775	0.675–0.888	0.000	0.915	0.833–1.006	0.066	*			0.819	0.665–1.008	0.059
UICC stage	1.886	1.144–3.107	0.013	5.531	3.023–10.119	0.000	7.117	3.699–13.925	0.000	2.029	0.906–4.544	0.085
CRP	1.045	1.019–1.071	0.001	1.024	1.002–1.046	0.033	1.028	1.007–1.050	0.009	*		

Abbreviations: OS, overall survival; FFS, failure free survival; D-FFS, distant failure-free survival; LR-FFS, localregional failure-free survival.* Not a significant factor.

Supplementary Table S3: Significant factors in multivariate analysis including lymphocyte CD28 expression of different endpoint in Cox regression model

Characteristics	OS			FFS			D-FFS			LR-FFS		
	HR	95% CI	*P value*	HR	95% CI	*P value*	HR	95% CI	*P value*	HR	95% CI	*P value*
BMI	0.775	0.675–0.888	0.000	0.915	0.833–1.006	0.066	*			0.819	0.665–1.008	0.059
UICC stage	1.886	1.144–3.107	0.013	5.531	3.023–10.119	0.000	7.177	3.699–13.925	0.000	2.029	0.906–4.544	0.085
CRP	1.045	1.019–1.071	0.001	1.024	1.002–1.046	0.033	1.028	1.007–1.050	0.009	*		

Abbreviations: OS, overall survival; FFS, failure free survival; D-FFS, distant failure-free survival; LR-FFS, localregional failure-free survival.* Not a significant factor.
